# Early-Stage Feasibility of a Mobile Health Intervention (Copilot) to Enhance Exacerbation-Related Self-Management in Patients With Chronic Obstructive Pulmonary Disease: Multimethods Approach

**DOI:** 10.2196/21577

**Published:** 2020-11-19

**Authors:** Yvonne JG Korpershoek, Tjitske Holtrop, Sigrid CJM Vervoort, Lisette Schoonhoven, Marieke J Schuurmans, Jaap CA Trappenburg

**Affiliations:** 1 Research Group Chronic Illnesses University of Applied Sciences Utrecht Utrecht Netherlands; 2 Nursing Science, Julius Center for Health Sciences and Primary Care University Medical Center Utrecht Utrecht Netherlands; 3 Imaging & Oncology University Medical Center Utrecht Utrecht Netherlands; 4 Education Center, UMC Utrecht Academy University Medical Center Utrecht Utrecht Netherlands

**Keywords:** mobile health, mHealth, mobile app, COPD, exacerbation, self-management, self-care

## Abstract

**Background:**

There is an emergence of mobile health (mHealth) interventions to support self-management in patients with chronic obstructive pulmonary disease (COPD). Recently, an evidence-driven mHealth intervention has been developed to support patients with COPD in exacerbation-related self-management: the Copilot app. Health care providers (HCPs) are important stakeholders as they are the ones who have to provide the app to patients, personalize the app, and review the app. It is, therefore, important to investigate at an early stage whether the app is feasible in the daily practice of the HCPs.

**Objective:**

The aim of this study is to evaluate the perceived feasibility of the Copilot app in the daily practice of HCPs.

**Methods:**

A multimethods design was used to investigate how HCPs experience working with the app and how they perceive the feasibility of the app in their daily practice. The feasibility areas described by Bowen et al were used for guidance. HCPs were observed while performing tasks in the app and asked to *think aloud*. The System Usability Scale was used to investigate the usability of the app, and semistructured interviews were conducted to explore the feasibility of the app. The study was conducted in primary, secondary, and tertiary care settings in the Netherlands from February 2019 to September 2019.

**Results:**

In total, 14 HCPs participated in this study—8 nurses, 5 physicians, and 1 physician assistant. The HCPs found the app acceptable to use. The expected key benefits of the app were an increased insight into patient symptoms, more structured patient conversations, and more tailored self-management support. The app especially fits within the available time and workflow of nurses. The use of the app will be influenced by the autonomy of the professional, the focus of the organization on eHealth, costs associated with the app, and compatibility with the current systems used. Most HCPs expressed that there are conditions that must be met to be able to use the app. The app can be integrated into the existing care paths of primary, secondary, and tertiary health care settings. Individual organizational factors must be taken into account when integrating the app into daily practice.

**Conclusions:**

This early-stage feasibility study shows that the Copilot app is feasible to use in the daily practice of HCPs and can be integrated into primary, secondary, and tertiary health care settings in the Netherlands. The app was considered to best fit the role of the nurses. The app will be less feasible for those organizations in which many conditions need to be met to use the app. This study provides a new approach to evaluate the perceived feasibility of mHealth interventions at an early stage and provides valuable insights for further feasibility testing.

## Introduction

### Background

Chronic obstructive pulmonary disease (COPD) is a highly prevalent disease and a major cause of mortality worldwide [1,2]. Exacerbations are important events during the course of COPD because they accelerate the decline in lung function [3], negatively affect quality of life [4,5], and lead to increased mortality and high socioeconomic costs [6,7]. Self-management is important to reduce the impact of COPD exacerbations on both patients and society. Self-management is defined as “an individual’s ability to detect and manage symptoms, treatment, physical and psychosocial consequences, and lifestyle changes inherent in living with a chronic condition” [8]. Over the past years, research has increasingly focused on exacerbation-related self-management interventions, as they have shown to have positive effects on quality of life and hospital admissions [9,10]. In this context, the use of mobile health (mHealth) is considered to be promising to engage patients in their own health and to change health behaviors [11-13].

Current eHealth and mHealth interventions often focus on telemonitoring strategies to reduce the impact of exacerbations [14-19]. Although positive outcomes were found for telemonitoring, the results are thus far inconclusive because of the poor quality of the studies and the heterogeneity among the studies [15,18,19]. The inconclusive results might also be explained by the lack of focus on enhancing self-management skills, as the decision-making process is mostly professional based. mHealth interventions aimed at facilitating, supporting, and sustaining self-management in patients with COPD have shown to improve quality of life and levels of activity; however, no firm conclusions could be drawn from them [13]. Recently developed mHealth interventions focusing on self-management have shown variable results. Farias et al [20] showed that using telehealth technologies to enhance adherence to a COPD action plan resulted in faster exacerbation recovery and decreased number of COPD-related emergency department visits and hospitalizations. Another mHealth tool that supports self-management of exacerbations showed no positive effects on exacerbation-free time, health status, and health care utilization [21]. However, given the proven effectiveness of self-management interventions in patients with COPD, it could be expected that mHealth interventions supporting patients in self-management can be effective in reducing exacerbation impact.

**Figure 1:**
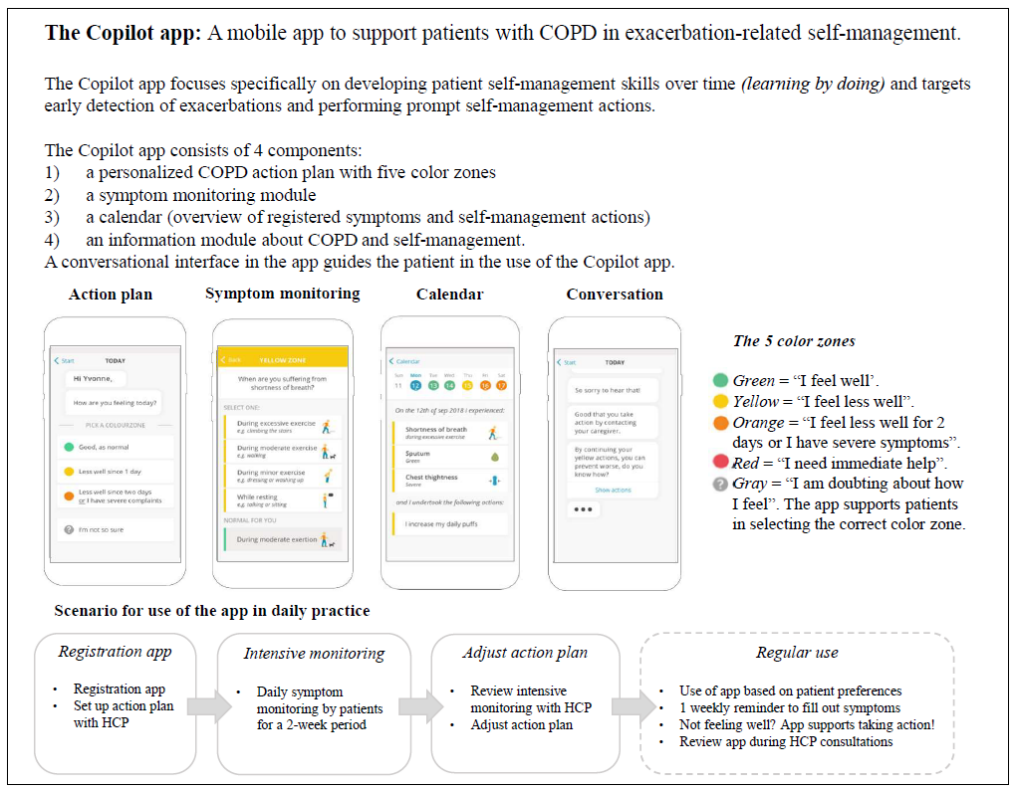
The Copilot app. COPD: chronic obstructive pulmonary disease; HCP: health care provider.

Recently, an evidence-driven mHealth intervention has been developed in the Netherlands to support patients with COPD (the end users) in exacerbation-related self-management—the Copilot app. The Copilot app is a mobile app that targets the early detection of exacerbations through self-monitoring and performing prompt actions by using individualized action planning. The Copilot app consists of 4 components: (1) a personalized COPD action plan, (2) symptom monitoring, (3) an overview of registered symptoms and undertaken self-management actions, and (4) an information module about COPD and self-management. The Copilot app focuses specifically on developing patient self-management skills over time (*learning by doing*) without real-time monitoring by health care providers (HCPs). Nevertheless, the HCPs are important stakeholders as the Copilot app requires a case manager role from HCPs. The role of the HCPs is to provide the app to patients, instruct patients on how to use the app, set up a personalized action plan together with a patient, and evaluate the patient’s condition based on registrations in the app during consultations. The app can be provided by HCPs across health care settings. The Copilot app was developed by following a user-centered design that included several phases of usability testing [22]. The usability of the Copilot app for patients was considered to be good [22]. More information about the Copilot app is provided in Figure 1.

An important next step is to investigate whether the Copilot app can work within the daily practice of HCPs by evaluating how HCPs perceive the feasibility of the Copilot app [23]. Evaluating feasibility within the daily practice of HCPs at an early stage helps to determine whether the Copilot app is appropriate for further testing and to identify what changes are needed and how they might occur [23]. Although patients with COPD and HCPs are both crucial stakeholders in feasibility evaluation, adequate personalization of the app and evaluation of the patient’s condition by HCPs is essential for safe and effective self-management by patients using the Copilot app [24,25]. Therefore, early feasibility evaluation in the daily practice of the HCPs should precede further longitudinal feasibility testing among patients. On the basis of this step, essential design input for optimization of the Copilot app can be generated and a substantial part of the feasibility problems in the daily care by HCPs can be eliminated before further testing.

### Objectives

The aim of this study is to evaluate the perceived feasibility of the Copilot app in the daily practice of HCPs.

## Methods

### Study Design

A feasibility study with a multimethods design was used to investigate how HCPs experience working with the first version of the Copilot app and how they perceive the feasibility of the app in their daily practice. On the basis of the work by Bowen et al [23], feasibility areas relevant for this early-stage feasibility evaluation were used for guidance. This study evaluates how HCPs react to the Copilot app (acceptability); the extent to which the Copilot app is likely to be used by HCPs (demand); the extent, likelihood, and manner in which the Copilot app can be used by HCPs as planned and proposed (implementation); the extent to which the app can be used by HCPs in their routine daily practice considering the available resources (practicality); and the extent to which the Copilot app can be integrated within the context of Dutch health care settings (integration) [23]. A one-time interactive session was conducted to observe how HCPs interacted with the app. HCPs were observed while performing tasks with the app and asked to *think aloud* to verbalize initial perceptions and feelings toward the app, clarify their process of decision making, and express experienced problems [26,27]. Afterward, a standardized usability questionnaire was used to investigate how HCPs perceived the usability of the app, and semistructured interviews were conducted with HCPs to further explore how they perceive the feasibility of the app. The study was conducted in primary, secondary, and tertiary care settings in the Netherlands from February 2019 to September 2019. The study was approved by the Medical Ethics Research Committee of the University Medical Centre Utrecht (18/831).

### Study Population

A purposive sample of HCPs who have a case manager role in COPD care was selected from primary, secondary, and tertiary care settings in the Netherlands. Case management was defined as physicians or nurses who provide ongoing and/or follow-up self-management support during patient consultations [28]. Eligible HCPs were pulmonologists, general practitioners, respiratory nurses, respiratory nurse specialists, physician assistants, and primary care nurses, with a minimum work experience of 1 year and employment at their present organization for at least one year. The 1-year cutoff point was considered relevant in providing meaningful insights into the feasibility areas. The maximum variation in profession and work setting was pursued to select a sample that adequately represents the HCPs providing self-management support in Dutch COPD care and to increase the likelihood of capturing different perspectives in the findings. On the basis of the guidance for evaluating the use of apps in general, a minimum of 10 HCPs were approached [27]. HCPs were included until saturation was reached for acceptability, demand, and implementation. Practicality and integration vary greatly across various health care organizations; therefore, it was not considered feasible to achieve saturation in these areas of focus using this study design. Therefore, data collected on practicality and integration are only described in this study.

### Recruitment and Informed Consent

HCPs were approached by email or telephone to participate in this study by 2 researchers (YK and TH). Four HCPs were approached based on their expressed interest during participation in previous studies related to the development of the app, whereas others were recruited through local COPD networks. The HCPs received an invitation to participate in the study by email. A reminder was sent after 1 week in case of nonresponse. In case of continued absence of a response, the HCPs were contacted within a week by phone to determine their interest to participate in the study. The HCPs willing to participate received further study information and an informed consent form. An appointment was scheduled at the place of employment of the HCPs, and the informed consent form was signed during the appointment. Recruitment started initially with 5 HCPs to initiate data analysis. Further recruitment was determined based on ongoing data analysis and data saturation for acceptability, demand, and implementation.

### Data Collection

Data were collected during a single, 1-hour session following a stepwise procedure. The stepwise procedure is described in the following sections.

#### Step 1: Interactive Session (Observations and the Think Aloud Method)

Before starting the interactive session, participants were informed about the study procedure and received further information about the Copilot app, the scenario for use of the app in daily practice, the intended role of both HCPs and patients, and the developmental stage of the app (Multimedia Appendix 1). Furthermore, the researcher walked through the 4 components of the app together with the participants. The participants were then asked to read a fictional patient case together with a set of tasks developed by the research team (Multimedia Appendix 2). These tasks focused on personalizing the action plan, adjusting the action plan, and evaluating the overview of registered symptoms and actions in the app. The participants were asked to perform these tasks in the app and were instructed and stimulated to *think aloud* while performing these tasks [26,27]. Almost all HCPs used the first version of the Copilot app. In the final interview, an updated version of the Copilot app was used, in which minor changes were made to improve usability. No major changes were made to the content and functions of the app; therefore, the influence of these changes on study outcomes was considered to be minor.

#### Step 2: The System Usability Scale

After working with the app, participants were asked to fill in the validated 10-item System Usability Scale (SUS) to obtain an overall view of the usability of the app for HCPs [29]. Each item was scored on a 5-point Likert scale, and all items were converted to a total score (range 0-100; a score >70 is considered to be acceptable) [29,30]. Although investigating usability was not the objective of this study, usability problems could have been experienced by participants who worked with the first version of the Copilot app. The perceived usability by HCPs was considered to be an important factor that could influence the adoption of the app by HCPs in daily practice and was therefore evaluated roughly in this study as well [31].

#### Step 3: Semistructured Interview

A semistructured interview was conducted to investigate perceptions toward the feasibility of the Copilot app in daily practice. A topic list was developed based on the 5 areas of focus and their related outcomes of interest [23]. Questions that were formulated by Bowen et al [23] to illustrate the areas of focus and the outcomes of interest were used as a basis for formulating interview questions. The topic list is further detailed in Multimedia Appendix 3. During and directly after the interviews, memos were created to describe observations, reflect on methodological issues, and capture initial thoughts related to theoretical concepts. Insights gained during the interviews were introduced in subsequent interviews, leading to data saturation.

#### Step 4: Baseline Questionnaire

After finishing the interview, participants were asked to fill in a questionnaire to collect baseline characteristics.

The stepwise procedure of data collection is further detailed in Table 1 and Multimedia Appendix 1.

**Table 1:** Stepwise data collection procedure related to the areas of focus and outcomes of interest.

Method and area of focus		Outcome of interest
**Step 1: interactive session**		
	Implementation^a^	Degree of execution of tasks and success or failure of execution of tasks
**Step 2: SUS^b^**		
	Acceptability^c^	Satisfaction with the app
	Demand^d^	Intention to use the app
	Implementation	Degree of execution of tasks
**Step 3: interview**		
	Acceptability	Satisfaction with the app, perceived appropriateness, and fit within the organizational culture
	Demand	Perceived demand and intention to use the app
	Implementation	Degree of execution of tasks, success or failure of execution of tasks, and factors affecting implementation ease or difficulty
	Practicality^e^	Expected benefits and burden for end users and ability of HCPs^f^ to carry out tasks in their routine daily practice
	Integration^g^	Perceived fit with local care infrastructure at the patient and organizational level and perceived sustainability at the patient and organizational level
**Step 4: baseline questionnaire**		Profession, age, work experience, size of organization, amount of patient consultations for COPD^h^ in a week, average time available for patient consultations, disease severity of patients with COPD in daily care, current use of written action plan, current use of mobile health

^a^Implementation: the extent, likelihood, and manner in which the Copilot app can be used by health care providers as planned and proposed.

^b^SUS: System Usability Scale.

^c^Acceptability: how the health care provider reacts to the Copilot app.

^d^Demand: To what extent is the Copilot app likely to be used by the health care provider.

^e^Practicality: To what extent the Copilot app can be used by health care providers in their routine daily practice considering the available resources.

^f^HCP: health care provider.

^g^Integration: to what extent can the Copilot app be integrated in Dutch primary, secondary, and tertiary care settings.

^h^COPD: chronic obstructive pulmonary disease.

All sessions were conducted by 1 researcher (YK or TH) who guided the interactive sessions and conducted the interview. The whole procedure was video recorded to observe the hand interaction of participants with the app and to audio record verbalizations during the interactive session and the interview. In addition, the researcher made notes on observed user problems and relevant verbalizations of the participants while they were working with the app. Before starting the data collection, 2 pilot sessions with professionals in COPD care were conducted to investigate whether participants understood the procedure and questions, to determine whether the questions resulted in relevant answers, and to determine whether the stepwise data collection procedure fitted in a 1-hour session. Findings of the pilot sessions were used to modify data collection procedures by reducing the set of tasks that HCPs had to perform, by adjusting the information about the app and study procedure, and by merging interview questions that resulted in similar answers. The results of the pilot sessions were not included in this study. Practical issues that arose during the study resulted in iterations in the data collection guideline (Multimedia Appendix 1).

### Data Analysis

Data from the interactive sessions and semistructured interviews were analyzed according to a thematic analysis as described by Braun and Clarke [32]. Video recordings were reviewed for usability issues and categorized according to the type of problem. All video recordings, including both the *think aloud* comments of the interactive sessions and the interviews, were transcribed verbatim. In total, 13 hours of video recordings were transcribed verbatim, resulting in 230 pages of transcription. Data analysis was supported by NVivo 11.0 software (QSR International Pty Ltd Version 11). The analysis took place in a cyclic process, alternating data analysis with data collection. Data were analyzed by 2 researchers (YK and TH) and were discussed with a third researcher (SV).

First, the 2 researchers independently read the transcripts to obtain an overall picture and noted initial ideas on relevant themes. Second, the transcripts were reread in more detail, and initial codes were linked to meaningful paragraphs by both researchers and discussed afterward to reach consensus. Next, identified codes were brought under potential themes and were reviewed for correspondence to the coded paragraphs, generating a thematic map of the analysis. Finally, potential themes were further refined, and clear definitions were generated for each theme. The third researcher was involved from the stage at which potential themes were reviewed, by coding a selection of data and participating in discussions on the final definition of themes. Interpretation of the data was discussed with experts in the fields of nursing science, self-management, and mHealth (JT, MS, and LS), which contributed to the credibility of the study [33]. Memo writing supported the data analysis process. The confirmability of the study was enhanced by peer review of the methodological quality by an external expert on qualitative research (SV) [33].

Data from the SUS and the baseline questionnaire were analyzed with SPSS 25.0 (IBM Corporation) using descriptive statistics.

## Results

### Baseline Characteristics of the Participants

A total of 14 HCPs (9 females and 5 males) participated in this study, including 8 nurses, 5 physicians and 1 physician assistant recruited from 5 primary, 7 secondary, and 2 tertiary care settings. The baseline characteristics of the participants are presented in Table 2. A total of 11 HCPs currently used written COPD action plans in their daily care to some extent, varying from barely using action plans to integrating action plans in regular care. A total of 10 HCPs had experience with the use of digital technology in COPD care; however, in most cases, technology was only used occasionally or within a study context. Maximum variation was achieved for profession, work setting, age, work experience, patient category most frequently seen by the HCP based on disease severity, number of patient consultations for COPD during a week, and organization size.

**Table 2:** Baseline characteristics of the participants.

ID	Age range (years)	Profession	Setting	Work experience (years)	Patient category^a^ based on GOLD^b^ stage	Patient consultations per week	Consultation duration (minutes)	Organization size^c^	Use of written action plan	Set up action plan (minutes)	Use of digital technology in COPD^d^ care
R01	30-39	Respiratory nurse	Hospital	2	3-4	20	30	251-1000	Yes	30	No
R02	50-59	Primary care nurse	General practice	30	2-3	15	45	26-50	No	N/A^e^	Apps
R03	50-59	Respiratory nurse	Hospital	6	3-4	28	20	>1000	Yes	15	Telehealth
R04	40-49	Respiratory nurse	Hospital	12	3-4	21	30	101-250	Yes	30	Telehealth
R05	30-39	Pulmonologist	Hospital	7	3-4	15	10	>1000	No	N/A	eHealth and apps
R06	50-59	Respiratory nurse specialist	Hospital	20	3-4	70	20	>1000	Yes	10	No
R07	40-49	General practitioner	General practice	7	1-2	4	10	101-250	Yes	10	eHealth
R08	60-69	Pulmonologist	Hospital	34	3-4	50	15	251-1000	Yes	20	eHealth
R09	40-49	Pulmonologist	Rehabilitation clinic	2	3-4	25	20	251-1000	Yes	0^f^	No
R10	50-59	Physician Assistant	Rehabilitation clinic	30	3-4	10	30	251-1000	Yes	15	No
R11	50-59	Primary care nurse	General practice	12	2-3	2	30	<10	Yes	10	eHealth
R12	40-49	General practitioner	General practice	10	1-2	4	10	11-25	Yes	30	eHealth and apps
R13	60-69	Respiratory nurse specialist	Hospital	14	2-3	20	30	>1000	Yes	30	Apps
R14	30-39	Primary care nurse	General practice	3	1-2	3	60	11-25	No	N/A	eHealth

^a^Patient category most frequently seen by the health care provider, based on Global Initiative for Chronic Obstructive Lung Disease stage.

^b^GOLD: Global Initiative for Chronic Obstructive Lung Disease.

^c^Organization size determined by the number of employees.

^d^COPD: chronic obstructive pulmonary disease.

^e^N/A: not applicable.

^f^The health care provider uses an action plan during consultations but does not set up an action.

The themes that emerged from this study are described in the following paragraphs and illustrated by quotes of the HCPs. Q references in the text refer to quotes of specific themes that are provided in the textboxes at the end of each paragraph. An overview of the themes is provided in Table 3. The themes are categorized based on the areas of focus, and the perceived benefits and risks of using the app in daily practice are described separately.

**Table 3:** Overview of the themes.

Area of focus			Themes
Acceptability of the app and perceived demand			High satisfaction User friendly Relevant for daily practice App fits well within the organizational culture High level of interest
Perceived benefits and risks of using the app in daily practice			A useful tool for patients to support self-management behavior Patients being the owner of the app could enhance patient control Improvement for patients compared with the use of written action plans More in-depth and structured patient conversations More insight into actual experienced symptoms More tailored treatment and self-management support Increase uniformity in self-management support by HCPs^a^ Enhance collaboration between HCPs within and across health care settings Concerns about the safety of the app Patients substituting HCP contact with the app The app could be distracting from interacting with patients Contribute to an increase in treatment burden
Factors that could influence the use of the app in daily practice			Patient skills, opportunity, and motivation The fit with the available time and workflow of HCPs The autonomy of the professional The focus of the organization on eHealth and self-management Costs associated with the use of the app Compatibility of the app with the current systems and eHealth initiatives
The extent to which the app can be used in current daily practice			The app being in line with the GDPR^b^ rules Clear instructions about the app for both patients and HCPs Sufficient time during consultations Approval of using the app within the organization Having a plan for implementation of the app Access to a fast Wi-Fi connection Good coordination between HCPs in collaborating organizations with regard to their roles and responsibilities A separate HCP portal would have added value Concerns about the privacy sensitivity of an HCP portal HCPs having access to the app could undermine or diminish self-management behavior of the patient
Integration of the app across Dutch health care settings			
	How to integrate the app into daily practice	The app is feasible to integrate into their daily practice and in already existing paths of care Flexibility in the moment of introducing the app to patients and evaluating the app Could be used as guidance during patient consultations Replace the use of written action plans for the app Sustainable in Dutch COPD^c^ care	
	The role of health care professionals	A shared responsibility between nurses and physicians The selection of eligible patients for the app should be the responsibility of HCPs Role in installing and personalizing the app Evaluate patient skills and use of the app	

^a^HCP: health care provider.

^b^GDPR: General Data Protection Regulation.

^c^COPD: chronic obstructive pulmonary disease.

### Acceptability of the App and Perceived Demand

When the HCPs were asked about their first impressions of the app, the HCPs spoke about the usability aspects of the app and the relevance of the app for daily practice. Overall, *high satisfaction* about working with the app was expressed by ratings of 7 or higher on a 10-point numeric scale. These high ratings were attributed to finding the app *user friendly* because of its ease of use, intuitive navigation, design simplicity, attractive layout, and receiving positive feedback when using the app (Q1). Although HCPs needed time to familiarize themselves with the app, working with the app was perceived to be easy to learn. The high level of satisfaction and the app being perceived as user friendly was also supported by an average score of 83.8 (SD 15.1) on the SUS, indicating good usability of the app.

Furthermore, almost all HCPs believed that the app would be *relevant for daily practice* (Q2)*.* HCPs recognized the content of the app, and particularly nurses found the app in line with the current self-management support. The calendar function in the app was considered to be most relevant for HCPs as it provided a compact, rapid, and clear overview of registered symptoms and actions. The action plan was largely in line with the current written action plans used by HCPs. The gray zone for decision support in determining the correct color zone (Figure 1) and the focus on personalizing the green zone based on registered symptoms were considered to have added value compared with written action plans. However, 8 HCPs preferred further tailoring of the yellow and orange zones (Figure 1) by adding more personalized signals for symptom deterioration. The information module was considered to be clear and relevant, although information could be presented more attractively and could be extended with more detailed information about self-management actions. Most HCPs were positive about the symptom monitoring module, as indicating symptoms fits well with how patients would describe their symptoms. A total of 3 HCPs found the symptom monitoring module to be more convenient than the currently used questionnaires in COPD care, such as the Medical Research Council dyspnea scale and the Clinical COPD Questionnaire [34,35], whereas 6 HCPs expressed a wish to add those questionnaires to the app.

A total of 11 HCPs expressed that the *app fits well within the organizational culture* as their organizations are open to digital innovations in health care because of the fit with the current environment in which health care digitalization is rapidly evolving. However, some organizations neither prioritize nor facilitate digital innovations yet. Almost all HCPs had a positive attitude toward using the app in daily practice because of opportunities to improve the quality of self-management support (Q3). When discussing colleagues’ attitudes toward using mHealth, 3 HCPs believed that physicians would be hesitant and resistant toward these innovations on account of time constraints in their profession.

The majority of HCPs expressed a *high level of interest* in using the app, scoring an 8 or higher on a 10-point numeric scale. HCPs explained these ratings by their personal interest and enthusiasm for innovations and the fit with their needs and demands for health care improvement, such as more structured self-management support for patients with COPD (Q4). All HCPs expressed having the intention to use the app in their own practice, except for 1 general practitioner who believed the app will not fit with the current workflow and related time constraints.

Quotes of HCPs (Q references in the text) related to acceptability of the app and perceived demand are provided in Textbox 1.

**Quotes related to acceptability of the app and perceived demand.**

Quotes of health care providers related to acceptability of the app and perceived demand:

- Q1: “I find it very useful, it is accurate and well-arranged and I find the lay-out very pleasant, actually. You’re not distracted by small letters or something on the side edge of the screen. I think it’s very clear.” (R10)
- Q2: “I believe the app is clinically relevant, yes. It is very much based on the questions we are asking nowadays, based on the Medical Research Council dyspnea scale and the Clinical COPD Questionnaire. I recognize those questions in the app, but it is more logically translated to the daily practice of the patients themselves in my opinion” (R14)
- Q3: “I think these sort of initiatives for a large part have the future and…and that it can make it easier for people, and that it will help. So I am very, very enthusiastic.” (R05)
- Q4: Interviewer: “How interested are you in using the app and why?” R11: “I think an 8 or 9, because self-management is returned to the person who has the disease. Also because currently, there is nothing. The culture is finally shifting as we discover, oh yes...the patient has to do it. What we have been doing with patients with diabetes for years already.” (R11)

### Perceived Benefits and Risks of Using the App in Daily Practice

The HCPs believed that there would mainly be benefits of using the app in daily practice at the patient, HCP, and organizational levels and only a few potential risks. Almost all HCPs found the app *a useful tool for patients to support self-management behavior* as it can help patients to create awareness of their stable symptoms and signals of symptom deterioration and could then support taking prompt and adequate actions. More than half of the HCPs believed that *patients being the owner of the app could enhance patient control* of the disease by becoming less dependent on their HCP and being in control when receiving support from HCPs across health care settings (Q5). The 4 HCPs explicitly mentioned that the app would be an *improvement for patients compared with the use of written action plans* as patients carry the app with them all the time and an app stays clean and readable.

The HCPs perceived many benefits of using the app for their own practice. Most importantly, 11 HCPs believed the app could lead to *more in-depth and structured patient conversations,* as the conversations would be more initiated by patients and, therefore, be more tailored to the specific needs and preferences of the patients (Q6). HCPs experience that patients often have difficulties with recalling experienced symptoms and performed actions and tend to underestimate or exaggerate their symptoms. The calendar in the app would provide *more insight into actual experienced symptoms* when patients are at home and prevents that HCPs have to dig for information. This could save valuable time and lead to more meaningful contact between HCPs and patients. The calendar could contribute *more tailored treatment and self-management support* by HCPs as the output could be used by physicians to evaluate medication treatment and by nurses to match patient needs with relevant self-management support (Q7-8). In addition, 5 HCPs believed that the app could *increase uniformity in self-management support by HCPs*. Nowadays, self-management support by HCPs is often inconsistent between HCPs within and across health care organizations. Some HCPs mentioned that the app could provide more guidance in providing self-management support and could also facilitate making clear agreements on which HCPs are assigned as contact persons for a patient (Q9). Moreover, most HCPs agreed that the app could be used by various HCPs throughout and across health care settings, including those without a case manager role, and could *enhance collaboration between HCPs within and across health care settings*. Although some HCPs expressed having good contact with HCPs in other health care organizations and clear agreements about their roles and responsibilities, others expressed experiencing limited collaboration between HCPs in primary, secondary, and tertiary care. Some HCPs expressed that the patient being the owner of the app would facilitate the patients themselves being able to show the agreements made about their treatment with an HCP in one setting to HCPs in other settings. This could result in more continuity of care (Q10). Most HCPs found it difficult to reflect on the potential benefits of the app on an organizational level. A few HCPs mentioned that the app could potentially reduce health care costs by preventing hospital admissions or reducing the duration of hospital admissions.

Most HCPs perceived limited risks associated with the use of the app in daily practice. However, some HCPs expressed *concerns about the safety of the app* as there is potential for making mistakes during manual registration of medication into the app. In addition, 1 nurse specialist was concerned about the misinterpretation of the color zones by patients. Furthermore, 1 general practitioner expressed that *patients substituting HCP contact with the app* could be a risk, as patients might be less likely to involve their HCP when they have the app to guide them. Especially during a stable phase, a patient might think that the HCP contact is redundant because their app indicates that all is well. Moreover, 1 pulmonologist felt that using *the app could be distracting from interacting with patients,* which could form an obstacle for the HCPs role (Q11). Finally, 1 HCP from tertiary care expressed that the app could *contribute to an increase in treatment burden* for patients as they are already treated in multidisciplinary teams with a variety of (digital) interventions (Q12).

Quotes of HCPs (Q references in the text) related to the benefits and risks of using the app in daily practice are provided in Textbox 2.

**Quotes related to the benefits and risks of using the app in daily practice.**

Quotes of health care providers related to the benefits and risks of using the app in daily practice:

- Q5: “I think patients will be more equipped to say: ‘These are my symptoms, and when if I have this then something really needs to be done’. And I think that especially in a situation when the orange zone is going towards red, that patients will be heard by HCPs, especially by the ones they don’t know well. A substitute general practitioner or emergency doctor or so...It gives them confidence that they know.” (R10)
- Q6: “Now you have a specific topic to discuss. Usually it’s small talk, but now patients will know in advance, ‘okay, we will discuss this.’ So also they will prepare in advance. So yes, I think it could be positive.” (R01)
- Q7: “I think, in the end, it could also save time because patients could clearly express their questions and problems. Based on that overview you could better target your consults and adequately meet patient needs.” (R03)
- Q8: “Look, if someone’s calendar is continuously ‘green’, you can say, ’well, that looks really good!, maybe we should cut back or adjust some medication. Let’s see if that is possible’. So that is all profit.” (R08)
- Q9: “Maybe more uniformity in how HCPs work, since the app would require a specific method of working. Currently, we all work in our own individual way (...) When you look at colleagues’ notes, you notice variation in reporting due to differences in focus. There is no consistency. So the app could stimulate that as well.” (R06)
- Q10: “By having the action plan on the phone the responsibility is given to the patient. When he comes into contact with other HCPs (...) you can say: ‘Look, the patient has it on his phone!’ And not only for us outpatient clinic but also for the nursing ward they can say: ‘Hey, what has the patient done? What happened?’” (R04)
- Q11: “You don’t have the time to fill out the app during a consult. In those 10 minutes you already have to type in a lot in the electronic patient file, and you have to talk to your patient and examine your patient as well. That doesn’t work. Looking patients in the eyes and listening to their lungs is most important for patients.” (R05)
- Q12: “If I put myself into the patients position, I think, ‘Now I have an app for exacerbations, and the food intake app and move monitor app. That is quite a lot.’ That’s the only thing that makes me hesitant, the treatment burden.” (R10)

### Use of the App by HCPs and Factors That Could Influence the Use of the App in Daily Practice

#### Observed Use of the App by HCPs

During the interactive session, all HCPs clearly understood the tasks they had to perform in the app and they were able to perform those tasks well. HCPs who had experience with written action plans expressed that setting up the action plan in the app corresponds with the current workflow of setting up a written action plan and could even improve this workflow. All HCPs were able to set up and adjust the action plan and review registered symptoms and actions in the calendar function of the app. Support from the researcher was needed when usability issues were observed. These issues were mostly related to the registration of medication, setting up the yellow zone of the action plan, and saving registrations.

#### Factors That Could Influence the Use of the App in Daily Practice

By asking HCPs about their perceptions toward using the app in their daily practice, HCPs reflected on factors that could facilitate or hinder using the app as intended at the level of patients, HCPs, and the organization. All HCPs believed *patient skills, opportunity, and motivation* influence the use of the app (Q13-14). The proliferation of mobile device use and improvement in the digital skills of patients were believed to facilitate the use of the app in a large patient population. However, low health literacy, avoiding confrontation with illness, limited digital skills, lack of access to the internet, and loss of interest in the app on the long term were considered to be threats for continued use.

*The fit with the available time and workflow of HCPs* was perceived to be an important factor that would influence the use of the app in daily practice. HCPs’ perceptions toward this fit were influenced by the traditional division between the roles of nurses and physicians. Most HCPs mentioned that the app fits within the available time and workflow of nurses as they already have an important role in providing self-management support (Q15). A total of 6 HCPs believed that the app does not fit within the available time and workflow of physicians as they have a main focus on medical treatment in the limited time they have available for patients (Q16). Two physicians even felt that using the app could result in more work as they would be obligated to focus on issues that normally would not come to light. For physicians, it was important to determine whether the app would improve their work efficiency (Q17). Furthermore, *the autonomy of the professional* in implementing innovations and scheduling extra time for consultations, if needed, was perceived to facilitate use of the app in daily practice (Q18). Moreover, 2 HCPs in primary care mentioned that primary care nurses might not feel comfortable with adding medication prescriptions to the app, which could hinder the use of the app as intended.

The *focus of the organization on eHealth and self-management* was considered to be an important facilitator for implementation of the app, as this would also facilitate the existence of innovation teams within organizations that could support HCPs in the use of digital innovations (Q19). Furthermore, some HCPs mentioned that *costs associated with the use of the app* would influence the use of the app in an organization, as innovations should not be too expensive and should ideally lead to a reduction in health care costs. Finally, some HCPs expressed that the *compatibility of the app with current systems and eHealth initiatives* in their organization is important, as a mismatch with current systems could hinder the use of the app in an organization (Q20).

Quotes of HCPs (Q references in the text) related to performance of tasks in the app and factors that could influence the use of the app in daily practice are provided in Textbox 3.

**Quotes related to performance of tasks in the app and factors that could influence the use of the app in daily practice.**

Quotes of health care providers related to performance of tasks in the app and factors that could influence the use of the app in daily practice:

- Q13: “We have a lot of older generations here. That could be complicated for them. But sometimes it takes me by surprise when someone of 90 has a tablet and iPhone. I am often surprised because you think, ‘Oh no, they will not do that’, and then all of the sudden, there is their phone!” (R04)
- Q14: “I do wonder if someone will actually work with it. Because there are also people who do not constantly want to be reminded about their illness and prefer to hide it.” (R01)
- Q15: “The content, we also work with that when we make plans. So it is in agreement with the work procedure we do without the app, what we do on paper now.” (R03)
- Q16: “Right now I am already thinking, for a doctor, for the consultation time available, this is too complicated, it takes too long. I am already thinking: ‘I have to continue. I don’t have that much time.’ Look, now I am not even talking with the patient.” (R05)
- Q17: “For patients, it would be an obvious improvement, but it will not directly be an improvement in efficiency for us.” (R05)
- Q18: ”To an extent, I am free to provide that kind of care of which I believe is necessary or has added value.” (R14)
- Q19: “Yes, we are actually ready for implementation at this time since we are currently working on all kinds of innovations, also innovations that support patients to be in control over their disease.” (R03)
- Q20: “I am not sure whether the app matches with the integrated care system we are currently using, since you have to focus a part of your consultation on the app where we normally follow our integrated care system that provides a certain structure for a consult.” (R14)

#### The Extent to Which the App Can be Used in Current Daily Practice

By asking HCPs how the app would fit within the current daily practice, 3 HCPs explained that the app could already be used in daily practice considering the available conditions, time, and resources. However, the majority of HCPs mentioned conditions that should be met before being able to use the app in daily practice. An important condition that should be met is *the app being in line with the General Data Protection Regulation (GDPR) rules,* as HCPs asked questions about privacy issues (Q21). In addition, some HCPs explained that the app should function technically well on various devices so that patients can use the device they prefer.

On patient and HCP levels, most HCPs expressed a need for *clear instruction about the app for both patients and HCPs,* such as written information or a demo about the app, to have sufficient knowledge about how the app should be used and to show patients how the app works (Q22). Especially for HCPs without the experience of working with written action plans, instruction on how to set up an action plan that includes medication prescription is important. Furthermore, for HCPs specifically, there should be *sufficient time during consultations* to be able to work with the app. On the basis of this study, HCPs expected to need approximately 30 min to install the app and to personalize the action plan for the patient. They believed this would probably take longer in daily practice as they would have to communicate with patients at the same time. Therefore, some HCPs explained that their consultation time should be extended. Most of these HCPs expressed that they would have the opportunity to schedule some extra time for consultations. Two HCPs emphasized the importance of embedding the app into current workflows to realize sustained use over time.

Many HCPs insisted that *approval of using the app within the organization* was considered to be an important condition that must be met before being able to use the app in daily practice (Q23). Having an organizational mandate to implement the app was considered to contribute to the allocation of time and resources. Overall, HCPs working in larger organizations believed acquiring management support to be more difficult and time consuming than HCPs working in smaller organizations. Moreover, 2 HCPs working in larger organizations expressed that *having a plan for implementation of the app* would be important to mobilize people in an organization to start working with the app. Furthermore, a practical issue mentioned was that organizations need to have *access to a fast Wi-Fi connection* to be able to quickly download and use the app in daily practice. Finally, HCPs emphasized the importance of *good coordination between HCPs in collaborating organizations with regard to their roles and responsibilities* in the use of the app. A total of 7 HCPs indicated that training and instructional material for HCPs across health care settings would help to create awareness about the app among HCPs and about their role in using the app (Q24).

On the basis of the question whether HCPs would prefer to have access to a separate HCP portal to set up an action plan and review patients’ registrations on their own computer, 8 HCPs expressed that *a separate HCP portal would have added value* for their daily practice. These HCPs, mostly physicians, believed that a separate portal would be more user friendly and efficient, would be helpful in preparing patient consultations, and would enable HCPs to review the app during consultations by telephone. Furthermore, it could reduce the administrative burden and could support collaboration between HCPs in an organization when the HCP portal is integrated into local information technology systems. *Concerns about the privacy sensitivity of an HCP portal* influenced the perceptions of the HCPs on having a separate portal. On one hand, 2 HCPs expressed that they would not feel comfortable if they had to work on the patient’s device itself, as this is a private device. On the other hand, some HCPs expressed that a connection with a separate portal or patient system could entail privacy risks as well. A separate HCP portal was less important for nurses. Three nurses argued that *HCPs having access to the app could undermine or diminish self-management behavior of the patient.* It could give patients the feeling of being monitored by their HCP, which emphasizes an external locus of control (Q25). Nonetheless, most HCPs perceived a separate HCP portal as an important condition that must be met to stimulate use of the app in daily practice.

Quotes of HCPs (Q references in the text) related to the extent to which the app can be used in current daily practice are provided in Textbox 4.

**Quotes related to the extent to which the app can be used in current daily practice.**

Quotes of health care providers related to the extent to which the app can be used in current daily practice:

- Q21: “It is important to know what will happen with the data. Will data be used for further research? What will happen with it? Will data be stored somewhere? Or will it only be available for patients themselves?” (R04)
- Q22: “Of course training for HCPs is necessary, but also to show patients the app. That you have an app with a an example of a patients and that you can show what you can do with it. Then the patient can decide to use the app or not. And yes.. an instructional flyer, with preferably a demo as well. Preferably on a desktop or on mobile device, that would work most handy because that is what they will work with.” (R02)
- Q23: “We have an agreement that new studies or implementations must be approved of by the management team. On the one hand, it always costs a little bit of time. On the other hand, you know when its approved then everybody has to abide by it. Then it will be supported by all location managers.” (R07)
- Q24: “We do collaborate with primary care, although this is not really translated into detailed care. If we were to implement the app, we also should inform primary care organizations about the app and that patients may come to their consults with the app instead of a written action plan.” (R06)
- Q25: “No, no...then you actually affirm or emphasize the external locus of control. The patient becomes passive, ‘I am being taken care of.’ And that is what we don’t want anymore!” (R06)

### Integration of the App Across Dutch Health Care Settings

#### How to Integrate the App Into Daily Practice

Overall, the HCPs from primary, secondary, and tertiary care settings felt that *the app is feasible to integrate into their daily practice and in already existing paths of care* as the app could be easily adapted to the specific context of health care organizations. The HCPs believed that the app could be introduced in annual COPD checkups in primary care; clinical care paths, outpatient follow-up care, and pulmonary rehabilitation programs in secondary care; and pulmonary rehabilitation programs in tertiary care (Q26). Most HCPs emphasized the importance of *flexibility in the moment of introducing the app to patients and evaluating the app* to be able to integrate the app into their workflow*.* Individual organizational factors, such as a specific path of care or division between HCP roles, would determine the specifics of how the app is to be integrated. The HCPs explained that the app *could be used as guidance during patient consultations.* HCPs currently using written action plans intended to *replace the use of written action plans for the app*. Nonetheless, for them, patient preferences and skills would determine whether a written action plan could be replaced by the app. Most HCPs felt that the use of the app would be *sustainable in Dutch COPD care* because of the fit within national COPD guidelines and current paths of care and their wish for more structured self-management support (Q27). However, 3 HCPs mentioned that the need for the app could decrease over time when the self-management skills of patients have been improved.

#### The Role of Health Care Professionals Toward Using the App

Although all HCPs indicated that working with the app best fits the role of the nurses because of their current role in providing self-management support, most HCPs considered the use of the app to be *a shared responsibility between nurses and physicians* (Q28). As nurses and physicians have a shared responsibility in the care for patients with COPD, this also applies to the use of the app in daily practice. Overall, introducing the app to patients and initiating and evaluating the action plan was considered to best fit the role of the nurses. However, some HCPs explained that physicians could have a role in prescribing and evaluating medication treatment in the action plan, which depends on the autonomy of the nurses with regard to medication prescription. HCPs expressed that reviewing the calendar of the app could be integrated in the consultations of both the nurses and physicians.

Most HCPs felt that *the selection of eligible patients for the app should be the responsibility of HCPs.* Although 4 HCPs expressed the intention to provide the app to all of their patients, most HCPs believed that not all patients will be eligible for the app. Therefore, they would select patients based on their assumptions about patient skills and motivation to use the app. A few HCPs explained that the motivation to use the app could be related to the severity of the disease. HCPs expected that patients with severe COPD and frequent exacerbations would be more motivated for exacerbation-related self-management compared with patients with an early stage of COPD who prefer to avoid confrontation with the disease. Therefore, most HCPs mentioned that they would provide the app to patients who frequently have exacerbations (Q29). Furthermore, most HCPs believed they would have an important *role in installing and personalizing the app* together with a patient, although 2 HCPs believed patients could do this initially by themselves. Finally, 6 HCPs mentioned that they would *evaluate patient skills and use of the app* so that they could provide support in using the app when needed, thereby guaranteeing safe and effective use by patients over time (Q30).

Quotes of HCPs (Q references in the text) related to integration of the app across Dutch health care settings are provided in Textbox 5.

**Quotes related to integration of the app across Dutch health care settings.**

Quotes of health care providers related to integration of the app across Dutch health care settings:

- Q26: ‘The app could actually fit in the current path of care we have in in this hospital, in which we also discuss self-management. It would fit with that. (...) It would be a new element to integrate, but it could be used as a supportive tool.” (R13)
- Q27: “The action plan can always be improved. And I think this app is an improvement. So in my opinion, it is future proof.” (R09)
- Q28: “The primary care nurses could start with filling out the patients name and symptoms. And if they do not feel comfortable with filling out medication, they can instruct the patient to bring the app with them to the yearly consult with the general practitioner who can fill out that part.” (R07)
- Q29: “The app can be useful for everyone, but I think it would be very useful for those patients that have clear symptoms and feel disabled, especially for those who frequently experience exacerbations. So if patients have frequent exacerbations I would be more inclined to offer the app to patients. However, I think I would have assumptions, unconsciously, about patients digital skills as well that could influence this decision.” (R07)
- Q30: “During the consult in which the symptom monitoring is evaluated you could as well evaluate how patients have used the app so far. You could let patients practice for example with how they could adjust the app. They have to learn how to use a new instrument.” (R6)

## Discussion

### Principal Findings

This early-stage feasibility study provides insight into the perceptions of Dutch HCPs with a case manager role in COPD care regarding the use of the Copilot app in daily practice. Overall, the HCPs were able to work with the app and found the app acceptable to use in daily practice. The app could be used as guidance during patient consultations and could replace the use of written action plans in COPD care. Many benefits and only a few risks were expected regarding the use of the app in daily practice at the patient, HCP, and organizational levels. The app was considered to best fit the role of the nurses. Physicians were expected to have a marginal role in working with the app because of time constraints and misfit with their workflow. Other key factors that could influence the use of the app were the autonomy of the professional, the focus of the organization on eHealth, costs associated with the app, and compatibility with the current systems used. There are various conditions that must be met to be able to use the app in daily practice. The level of importance of these conditions varied between professions and contexts and may be attributed to organizational factors or fundamental differences in needs between physicians and nurses. The app was considered to be feasible to integrate into existing care paths of primary, secondary, and tertiary health care settings. Individual organizational factors must be taken into account when integrating the app in daily practice.

Some of the findings of this study are in line with those of other studies. Two recent studies focusing on the adoption of mHealth by HCPs also identified usefulness, ease of use, perceived benefits, autonomy of the professional, and integration with other systems as facilitators for the adoption of mHealth by HCPs [31,36]. Similar to our results, these studies considered disruption to workflow, lack of time, increased workload, cost issues, and privacy and security issues as key adoption barriers [31,36]. Gagnon et al [31] pointed out that the use of mHealth could be disruptive during visits as it could influence the interaction between patients and health care professionals; this was identified in our study as well and was perceived as a risk for the use of the app in daily practice. Furthermore, a study focusing on the adoption of new technology by physicians found that high initial physician time costs, uncertain financial benefits, and lack of electronic exchange between systems were key physician-related barriers [37]. These studies indicate that nurses may hold the key to successful implementation of the Copilot app because of their role, the fit with their workflow and available time, and numerous advantages for their daily practice. Moreover, these studies strengthen our findings on the importance of meeting specific conditions to use the app in daily practice. According to our results, a separate portal for HCPs and integration with current systems could potentially facilitate the use of the app, especially for physicians. However, HCPs’ perspectives toward system integration differ, which was also observed in conversations with HCPs during the development of the Copilot app [22]. On the basis of the literature, it could be expected that interoperability is important for integration of the app across health care settings [31].

The findings of this study show that factors influencing future implementation and integration of the app into health care organizations are context dependent. Recently, much emphasis has been placed on the importance of taking into account the context in intervention research aiming at changing behaviors, to increase the likelihood of developing appropriate, implementable, effective, and sustainable interventions [38]. On the basis of HCPs’ perceptions that the app is feasible to implement and integrate into Dutch health care organizations, taking context into account in the development of the app seemed to have resulted in sufficient flexibility in the design of the app to work across a range of contexts [22,38].

### Strengths and Limitations

A strength of this study was the maximum variation in settings and HCPs resulting in a broad range of perspectives, thereby increasing the transferability of our findings to similar settings in the Netherlands [33]. Furthermore, the credibility and confirmability of this study were enhanced by using data and researcher triangulation [33]. The feasibility framework described by Bowen et al [23] ensured that feasibility was evaluated by considering several important areas of focus to determine if the app can work within the constraints of daily practice. Although not the focus of data saturation, data collected on integration and practicality gave a general impression of contextual differences on how to integrate the app and which HCP role is perceived to be most suited.

A limitation of this study was the variation in the course of the interactive sessions and interviews because of time constraints and unforeseen circumstances within the HCPs’ workflow. In some cases, this resulted in limited in-depth interviews and underexposure of some topics. However, systematic reflection on these methodological issues and subsequently adapting the guideline of the session resulted in more in-depth data collection as the study proceeded. Furthermore, a relatively large part of the study population had experience with digital technology to some extent. This may have resulted in a more positive perception toward the use of technology as familiarity with mHealth and technologies in general is considered to facilitate the adoption of mHealth [31]. Finally, this early-stage feasibility study evaluated the Copilot app within an artificial context, consisting of 1 interactive session with a fictional patients’ case. It could be discussed whether perceptions of feasibility would be different in the case of actual implementation of the app in the daily practice of the HCPs. Nonetheless, HCPs have experienced working with the app by simulating the use of the app in daily practice.

### Implications for Practice and Future Research

The findings of this study are important for HCPs in COPD care and for researchers focusing on the development and evaluation of mHealth interventions. The study shows that the Copilot app is considered to be relevant and acceptable to use in the daily practice of the HCPs. The app could result in various benefits for patients, HCPs, and health care organizations and has high potential for successful implementation and integration across Dutch health care settings. Important lessons can be learned from this study with regard to practicality, which we described in this study as conditions that have to be met to use the app in daily practice. To use the app in daily practice, it is important that clear instruction about the use of the app is provided to both patients and HCPs, that there is sufficient time during consultations, and that approval to use the app within organizations is realized. In addition, good coordination about the use of the app between HCPs in collaborating organizations is needed. Adequate training and support for HCPs regarding the use of the app is important for implementation and integration of the app in daily practice, as using the app requires behavior change from HCPs. Essential in changing HCPs’ behaviors is that they have the capability, motivation, and opportunity to use the app in daily practice [39]. Training and support should therefore focus on motivating HCPs to use the app and enhancing HCPs’ knowledge and skills needed to use the app, with a specific focus on the use of action plans [40]. Finally, a separate portal for HCPs is an important condition that must be met in some organizations to stimulate the use of the app in daily practice. Contextual factors across health care settings will determine the specific conditions that should be met to be able to use the app in daily practice.

For researchers and developers focusing on the development and evaluation of mHealth interventions, this study provides insight into a new approach to evaluate the feasibility of mHealth interventions at an early stage. This approach has been shown to be a thorough and relatively quick way to investigate perceptions toward feasibility. The methods used in this study provided rapid insight into influencing factors and conditions regarding feasibility, thereby allowing researchers and developers to adapt mHealth interventions by moving backward or forward quickly. Evaluating feasibility at an early stage helps to determine whether mHealth interventions are appropriate for further feasibility testing with end users over a longer time period.

Further research on the Copilot app should focus on longitudinal feasibility testing of the Copilot app with both patients and HCPs to investigate the delivery and acceptability of the intervention, compliance with the intervention, and recruitment of patients and to investigate limited efficacy. In a next phase, the effect of the Copilot app on relevant patient outcomes and health care use should be evaluated. This evaluation should include an assessment of how context influences the effectiveness of the app [38]. Understanding how the app relates to context is critical to understand how the app works and for whom, what influences implementation success and failure, whether the app can be successfully adapted or scaled-up from one context to another, and to what extent effects could be generalized to other contexts [38]. To achieve this, a thorough process evaluation using qualitative and quantitative methods from a system lens is recommended [38,41,42].

### Conclusions

This early-stage feasibility study shows that the Copilot app is feasible to use in the daily practice of Dutch HCPs and is considered to best fit the role of the nurses. The app is perceived to be acceptable to use and relevant for the daily practice of HCPs. The app can be used as guidance during patient consultations and could replace the use of written action plans in COPD care. Many benefits and only a few risks were expected regarding the use of the app in daily practice at the patient, HCP, and organizational levels. The app will be less feasible in organizations where relatively many conditions need to be met. The app is considered to be feasible to be integrated into primary, secondary, and tertiary health care settings in the Netherlands. Individual organizational factors must be taken into account when integrating the app in daily practice. This study provides a new approach to evaluate the perceived feasibility of mHealth interventions at an early stage and provides valuable insights for further feasibility testing. Future research should focus on longitudinal feasibility testing of the Copilot app by both patients and HCPs.

## Appendix

Multimedia Appendix 1 Stepwise procedure of data collection.

Multimedia Appendix 2 Fictional patient case and assignment for health care providers.

Multimedia Appendix 3 Topic list for the semistructured interview.

## Abbreviations

COPD: chronic obstructive pulmonary disease
GDPR: General Data Protection Regulation
GOLD: Global Initiative for Chronic Obstructive Lung Disease
HCP: health care provider
mHealth: mobile health
SUS: System Usability Scale
