# Supplementary material for: Early-Stage Feasibility of a Mobile Health Intervention (Copilot) to Enhance Exacerbation-Related Self-Management in Patients With Chronic Obstructive Pulmonary Disease: Multimethods Approach
Source: JMIR Form Res. 2020 Nov 19;4(11):e21577. doi: 10.2196/21577 (PMC7714642; doi:10.2196/21577)
Supplement: Multimedia Appendix 1 [file formative_v4i11e21577_app1.pdf]

Multimedia Appendix 1

Stepwise procedure of data collection

Guideline for stepwise procedure of data collection

**Introduction**	
Introduce study aim	• Evaluation of perceived feasibility of the Copilot app in the HCPs daily practice.<br>• This is an early feasibility study meaning that the perceived fit of the app in daily practice will be evaluated.<br>• The session consists of three parts:<br>o An interactive session → HCP works with the app using a patient case;<br>o Filling out System Usability Scale (SUS)<br>o An interview → HCP reflects on the interactive session and on perceptions towards feasibility of the app in daily practice;<br>o Filling out questionnaire on participant characteristics.<br>• Estimated duration: interactive session & SUS (20 minutes); interview (20 minutes), questionnaire (5 minutes) and rounding off session (5 minutes).
Describe aim of video recording	To observe the hand interaction of HCPs while working with the app and to audio record verbalizations during the interactive session and the interview.
Collect informed consent & questions	• HCPs are asked to sign the informed consent form in duplicate.<br>• Potential questions are answered.
Questions to ask before starting session	• HCPs are asked about their current experiences with COPD action plans.<br>• HCPs are asked about their experiences with using technology in COPD care.
**Introduction of the Copilot app *[by showing the app to HCPs]***	
Explain the intended use of the app	The Copilot app:<br>• Is developed for patients with COPD;<br>• Helps patients to recognize fluctuations in symptoms and to take prompt self-management actions;<br>• Contains a symptom diary, a personalized action plan, an overview of registered symptoms and actions (calendar) and information about COPD and self-management *[show components in app];*<br>• Is specifically intended to be used for self-monitoring of symptoms by patients. It is explicitly not a monitoring system for professionals (this is a difference compared to systems that focus on monitoring by HCPs).
Explain color zones of the action plan	• **Green** = "I feel well'. Current symptoms are 'normal'. The app supports patients in creating awareness on their 'normal' symptoms, their maintenance therapy and how they can stay in the green zone.<br>• **Yellow** = "I feel less well". There is an increase in one or more symptoms. The app supports patients to take action according to the mutual agreed actions between patient and HCP included in the action plan.

(Continued)

(Continued)

Explain color zones of the action plan	• **Orange** = "I feel less well for 2 days or I have severe symptoms". The app supports patients to take action according to the mutual agreed actions between patient and HCP included in the action plan.<br>• **Red** = "I need immediate help". Symptoms are life threatening. The app supports calling for immediate help.<br>• **Gray** = "I am doubting about how I feel". The app supports patients in selecting the correct color zone.
**Explain the scenario 'how to use the app'**	
1. Registration of app and personalization of action plan	During a patient consultation, the HCP and/or patient downloads the app onto the mobile device of the patient and together they personalize the action plan.
2. Intensive symptom monitoring	To determine what are the patients 'normal' symptoms, the patient intensively monitors his/her symptoms for two weeks by turning on the 'symptom diary'.
3. Reviewing and adjusting the action plan	After two weeks of daily symptom monitoring, the action plan can be adjusted. How and when adjustment of the action plan takes place is flexible, meaning this could be decided by individual health care organizations.
4. Regular use (self-monitoring)	One a week, the patient is actively asked how he/she feels by receiving a notification on the mobile device. Besides this request, the patient can register symptoms any time they want based on individual preferences.
5. Regular use (actions)	Dependent on the color zone, the patient receives tailored support regarding self-management actions.
6. Evaluating app	Using the calendar, the registered symptoms and actions are evaluated during patient consultations. If necessary, a new period of intensive monitoring can be deployed.
**Explain the role of patients and HCPs**	
The patient role	• Is the owner of the app, shows the app to all relevant HCPs involved;<br>• Makes a personalized action plan together with an HCP;<br>• Registers symptoms and undertaken actions;<br>• Uses the information module to search for information about COPD, exacerbations and self-management.
The HCP role	• Personalizes the action plan together with the patient;<br>• Specifically focusses on filling out the green zone: what symptoms are 'normal' for the patient;<br>• Evaluates the calendar with the patient during consultations → evaluation of registered symptoms and actions; if necessary, adjustment of action plan.
**Explain current developmental stage of app**	
	• The app HCPs work with is the first prototype of the app;<br>• Researchers are currently exploring potential ways to develop a portal for HCPs to be able to personalize and review the app;<br>• Usability testing with patients and HCPs has been completed. Some usability issues are not yet resolved in this current prototype. The researcher emphasizes the primary focus on evaluating perceptions towards feasibility of the app instead of focusing on usability issues.

(Continued)

(Continued)

**Explain expectations during interactive session**	
	• HCPs are asked to read the fictional patient case *(see Multimedia Appendix 2)* which focuses on an initial consultation in which the app will be personalized and a follow-up consultation;<br>• HCPs are asked to conduct several tasks within the app using the patient case, tasks are in line with how the HCP would work with the app in daily practice;<br>• HCPs are asked to 'think aloud' during the interactive session → explain feelings, thoughts, etc.
Part 1:<br>Initial consultation	Show patient case and explain:<br>• Pretend that Mr. Janssen* is sitting ahead of you for his first consultation;<br>• Set up an initial action plan based on the information provided in the patient case about symptoms and actions.
Part 2:<br>Follow-up consultation	Show patient case and explain:<br>• Pretend Mr. Janssen returns to see you after several weeks;<br>• Mr. Janssen has registered his symptoms over this time period;<br>• Adjust the action plan based on the information provided in the patient case about registered symptoms during the past weeks.
**Hand out System Usability Scale (SUS)**	
**Conduct semi-structured interview *(see topic list in Multimedia Appendix 3)***	
**Collect participant's baseline characteristics by handing out the questionnaire**	

Abbreviation: HCP: health care provider. *Mr. Janssen is a fictional patient.
