# Supplementary material for: Early-Stage Feasibility of a Mobile Health Intervention (Copilot) to Enhance Exacerbation-Related Self-Management in Patients With Chronic Obstructive Pulmonary Disease: Multimethods Approach
Source: JMIR Form Res. 2020 Nov 19;4(11):e21577. doi: 10.2196/21577 (PMC7714642; doi:10.2196/21577)
Supplement: Multimedia Appendix 2 [file formative_v4i11e21577_app2.pdf]

Appendix 2

Fictional patient case and assignment for health care providers

Textbox 1. Patient case: Initial consultation with 'Mr. Janssen'*

Aim: Installing and personalizing the action plan with Mr. Janssen.

Patient information for initial consultation:

- Mr. Janssen, 63 years of age, COPD GOLD II/D, knows you as HCP.
- Mr. Janssen has experienced frequent exacerbations during the past year, including being hospitalized four months ago.
- 'Normal' COPD symptoms include:
    - Shortness of breath (during light activity)
    - Coughing (occasionally)
    - Sputum (a little)
    - Fatigue (a bit tired)
- Normal treatment <u>(green zone):</u>
    - Long-acting bronchodilator: Spiriva Respimat 1x 2 doses per day
    - Inhaled corticosteroids: Foster doses aerosol 2 x 1 doses per day
- Actions to be taken in case of symptom deterioration <u>(yellow zone)</u>:
    - Extra medication:
        - Atrovent doses aerosol 3-4 doses per day
    - "I divide my energy/activities throughout the day"
    - "I conduct by breathing exercises"
    - 'In case of questions, I contact my HCP"
- Mutual agreements in <u>orange zone:</u>
    - Call your contact person (contact person(s) can be filled out by the HCP)

*Mr. Janssen is a fictional patient.

Textbox 2. Patient case: Follow-up consultation with 'Mr. Janssen'* after several weeks

Aim: Evaluate registered symptoms/actions and adjust action plan

Patient information for follow-up consultation:

Mr. Janssen has registered his symptoms every day for the past two weeks. He also registered the actions he undertook when feeling less well. Mr. Janssen is visiting you and shows which symptoms he had experienced. After evaluating this overview, you can tell the symptoms he indicated 'normal' do not correspond to symptoms of the green zone that were initially filled out in the action plan. At that time, Mr. Janssen did not exactly know which symptoms are normal for him. The registered symptoms indicate that Mr. Janssen coughs often (instead of coughing occasionally) and is very tired (instead of a bit tired). Furthermore, Mr. Janssen has experienced that he wheezes occasionally. Based on this information, you agree with Mr. Janssen that he can increase the dosage Foster from 2x1 to 2x2 per day during the green zone.

*Mr. Janssen is a fictional patient.

Textbox 3. Assignment for HCPs

Tasks to conduct within the app using the patient case of 'Mr. Janssen'*

1. **Initial consultation:**
    1) Read the information in the patient case related to the initial consultation.
    2) Register the Copilot app according to the onboarding conversation of the app.
    3) Individualize all color zones of the action plan according to the provided patient information.

2. **Follow-up consultation:**
    1) Read the information in the patient case related to the follow-up consultation.
    2) Adjust Mr. Janssen's normal symptoms (green zone) [based on the provided patient information]
    3) Adjust the medication in the green zone.
    4) Using the calendar, evaluate the registered symptoms and actions with Mr. Janssen.
    5) Take a look at the information module in the app. You can use the module to educate patients about COPD and the importance of self-management.

*Mr. Janssen is a fictional patient.
