# Supplementary material for: Early-Stage Feasibility of a Mobile Health Intervention (Copilot) to Enhance Exacerbation-Related Self-Management in Patients With Chronic Obstructive Pulmonary Disease: Multimethods Approach
Source: JMIR Form Res. 2020 Nov 19;4(11):e21577. doi: 10.2196/21577 (PMC7714642; doi:10.2196/21577)
Supplement: Multimedia Appendix 3 [file formative_v4i11e21577_app3.pdf]

Appendix 3

Topic list semi-structured interview

Parameter	Outcome of interest	Questions for HCP
Acceptability	Satisfaction with the app	• What is your first impression of the app?<br>• How did you experience working with the app? (What did you find easy to use? What did you find difficult to use?)<br>• On a ten-point numeric scale, how satisfied were you about working with the app? (why this rating? To increase this rating, what should be changed?)<br>• What is your opinion about the content of the app? Please explain<br>• Would you advice the app to your colleagues? Please explain why.
	Perceived appropriateness	• How usable is the app in your daily practice?<br>• How usable is the app for your patients?
	Fit within the organizational culture	• To what extent does the app fit within the culture of your organization?<br>• To what extent is your organization willing to use an app to support self-management in patients with COPD?
Demand	Perceived demand of the app	• On a ten-point numeric scale, to what extent are you interested in using the app in your daily practice? (why this rating? To increase this rating, what should be changed?)<br>• To what extent does the app fulfill your needs/wishes regarding improvement of COPD care?<br>• What would it mean for your daily practice when you start using the app?
	Intention to use the app	• On a ten-point numeric scale, how motivated are you to use the app in daily practice? (why this rating? To increase this rating, what should be changed?)[a]<br>• How would you most likely use the app in your daily practice?<br>• How do you think the app would be used in your organization?<br>• Are there elements of the app that would be used more likely than other elements?
Implementation	Degree of execution of tasks	• To what extent did you succeed in working with the app based on the tasks? Please explain. [b]<br>• Were you able to individualize the action plan in the app like you would normally do using a written COPD action plan?
	Success/failure of execution of tasks	• What went well during performance of tasks? [b]<br>• What was hard to handle during performance of tasks? [b]
	Factors affecting implementation ease or difficulty	• Which factors in your daily practice might hinder the app being used as intended? (HCP level/organizational level)<br>• Which factors in your daily practice could facilitate the app being used as intended? (HCP level/organizational level)

(Continued)

(Continued)

Practicality	Expected benefits and burden for HCPs	• To what extent can the app be used in your daily practice considering the available resources, time and commitment?<br>• In your opinion, what are expected benefits of using the app?<br>- *For your workflow*<br>- *For your interaction with patients*<br>- *For your organization*<br>• In your opinion, what are expected disadvantages or potential risks of using the app?<br>- *For your workflow*<br>- *For your interaction with patients*<br>- *For your organization*
	Ability of HCPs to carry out tasks in their routine daily practice	• To what extent do you feel able to use the app in your daily practice, considering the available resources, if the app was currently available?<br>• To what extent are the right conditions present to be able use the app?<br>• Which resources/conditions need to be met in order to use the app within your organization?<br>• What are your expectations regarding the time it takes to: 1) individualize the app? 2) adjust and evaluate the app in a follow-up consultation?
Integration	Perceived fit with local care infrastructure at patient and organizational level	• To what extent does working with the app fit within your routine daily practice?<br>• How do you think the app can be integrated into your daily practice?<br>• How do you think the app can be integrated into the daily practice of your colleagues? Which HCPs should be involved in working with the app? In which patient consultations could the app be integrated?<br>• How do you think the app can be integrated into your organization?<br>• To what extent does the app fit within the current collaboration between primary, secondary and tertiary care?
	Perceived sustainability at patient and organizational level	• In your opinion, which changes need to occur in order to integrate the app into your daily practice? And into your organization? And within current collaboration with other health care organizations?<br>• To what extent will the app be sustainable to use within your organization?

[a] HCPs were asked in the baseline questionnaire to fill out their intention to use the app in daily practice. Therefore, this question was often omitted during the interview; [b] Performance of tasks was also observed by the researcher during the interactive session. If needed, this was further evaluated during the interview.

Abbreviation: HCP: health care provider.
